# Increased expression and local accumulation of the Prion Protein, Alzheimer Aβ peptides, superoxide dismutase 1, and Nitric oxide synthases 1 & 2 in muscle in a rabbit model of diabetes

**DOI:** 10.1186/1472-6793-10-18

**Published:** 2010-09-06

**Authors:** Claudine L Bitel, Yicheng Feng, Nizar Souayah, Peter H Frederikse

**Affiliations:** 1 Department of Pharmacology and Physiology, UMDNJ-New Jersey Medical School, 185 S. Orange Ave. Newark, NJ 07103, USA; 2 Rutgers-UMDNJ Integrative Neurosciences Program, 185 S. Orange Ave, Newark, NJ, 07103, USA; 3 Department of Neurology and Neurosciences, UMDNJ-New Jersey Medical School, 185 S. Orange Ave. Newark, NJ 07103, USA

## Abstract

**Background:**

Muscle disease associated with different etiologies has been shown to produce localized accumulations of amyloid and oxidative stress-related proteins that are more commonly associated with neurodegeneration in the brain. In this study we examined changes in muscle tissue in a classic model of diabetes and hyperglycemia in rabbits to determine if similar dysregulation of Alzheimer Aβ peptides, the prion protein (PrP), and superoxide dismutase 1 (SOD1), as well as nitric oxide synthases is produced in muscle in diabetic animals. This wild-type rabbit model includes systemic physiological expression of human-like Alzheimer precursor proteins and Aβ peptides that are considered key in Alzheimer protein studies.

**Results:**

Diabetes was produced in rabbits by injection of the toxic glucose analogue alloxan, which selectively enters pancreatic beta cells and irreversibly decreases insulin production, similar to streptozotocin. Quadriceps muscle from rabbits 16 wks after onset of diabetes and hyperglycemia were analyzed with biochemical and *in situ *methods. Immunoblots of whole muscle protein samples demonstrated increased PrP, SOD1, as well as neuronal and inducible Nitric oxide synthases (NOS1 and NOS2) in diabetic muscle. In contrast, we detected little change in Alzheimer Aβ precursor protein expression, or BACE1 and Presenilin 1 levels. However, Aβ peptides measured by ELISA increased several fold in diabetic muscle, suggesting a key role for Aβ cleavage in muscle similar to Alzheimer neurodegeneration in this diabetes model. Histological changes in diabetic muscle included localized accumulations of PrP, Aβ, NOS1 and 2, and SOD1, and evidence of increased central nuclei and cell infiltration.

**Conclusions:**

The present study provides evidence that several classic amyloid and oxidative stress-related disease proteins coordinately increase in overall expression and form localized accumulations in diabetic muscle. The present study highlights the capacity of this wild-type animal model to produce an array of hallmark pathological features that have also been described in other muscle diseases.

## Background

Muscle disease has been linked with aging as well as metabolic conditions, which prominently includes diabetes [1-4]. In diabetes, weakness, wasting, and pain have been cited which commonly occur in quadriceps muscles. Muscle disease has been studied in a variety of conditions including animal models of high dietary cholesterol [5], pathological response to the drug chloroquine [6,7], and inclusion body myositis [8]. Each of these muscle conditions has been shown to produce localized accumulations of PrP and Aβ peptides, as well as superoxide dismutase 1 (SOD1) and Nitric oxide synthases 1 and 2 (NOS1: neuronal nNOS; and NOS2: inducible iNOS) [9,10]. These proteins are more often thought of in the context of neurodegenerative diseases (PrP, mad cow disease; Aβ, Alzheimer disease (AD); and SOD1, Amyotrophic lateral sclerosis (ALS)/Lou Gehrig's disease). However, these proteins have also been linked with diabetes. For example, strong associations between AD and diabetes has led to use of the term 'Type III diabetes' for AD [11]. More recently, extensive epidemiological data linking AD and diabetes are now supported by direct mechanistic links between Aβ peptide action and insulin receptor dysfunction in cells. Aβ peptides form small diffusible oligomers that can interact directly with insulin receptors (IR) on cell surfaces. This can lead to coordinate endocytosis of Aβ/IR complexes, and intracellular co-localization, which has been shown in cultured cells. Moreover, this process has also been linked with insulin resistance [12,13]. Evidence for a role for these amyloid disease proteins in muscle disease also comes from transgenic models, where muscle-specific expression of PrP, Aβ or SOD1 also produced localized accumulation. In addition, these muscle disease models also showed evidence of central nuclei and cell infiltration [14-19].

Deleterious activities attributed to these disease proteins is linked with an ability to produce oxidative stress, and also to form oligomers and aggregates in brain, muscle, and lens, as well as pancreas during onset and progression of Type II diabetes [20-25]. Diabetes and hyperglycemia increase systemic and tissue-specific oxidative stress loads. Consistent with this, increases in glucose oxidation products have been measured in muscle as well as in the circulatory system in diabetic animals [26,27]. Classic oxidative stress responses include stimulation of stress signaling pathways and activation of genes linked with stress and also cell proliferation, in part due to the activation of AP1 and NFκB stress-responsive transcription factors [28-30]. Previously, our laboratory also demonstrated PrP and Alzheimer Aβ Precursor Protein (AβPP) gene expression increase significantly during a response to oxidative stress [31-33]. Stress in tissues that include muscle can be aggravated by diabetes and high glucose that contributes to increased amyloid and oxidative stress-related protein expression and formation of protein deposits as well as producing further oxidative stress. In addition, stress signaling can affect normal cell migration and trophic responses [31,34].

In the present study we examined expression of PrP, AβPP and Aβ, SOD1, NOS1 and NOS2 in muscle in 4 month-old wild-type (wt) rabbits induced to become diabetic and hyperglycemic with alloxan. This model has been used to model diabetic complications in >500 studies over the past 50 yrs. Unlike mice and rats, wt rabbits produce Aβ peptides with the same sequence as humans. Mouse and rat Aβ has lower metal affinity that limits its ability to form oligomers and also to produce oxidative stress [20-23]. Like streptozotocin, alloxan is a toxic glucose analogue that selectively enters pancreatic β-cells *via *Glut2 glucose transporters, which are not expressed in muscle [35-37]. Alloxan forms toxic reactive oxygen species in pancreatic β-cells, and irreversibly decreases insulin production. Consistent with these activities, alloxan has a short half-life (~1.5 min), and is cleared quickly. Here, we examined quadriceps from diabetic and normal control rabbits 16 weeks after onset of diabetes and hyperglycemia. Studies of diabetic complications in other organs using this model indicated a time frame of 4-6 mos produced substantial effects [38,39]. We identified substantial increases in PrP, SOD1, NOS1, and NOS2 expression on immunoblots of total muscle protein samples from diabetic animals, and ELISA assays measured several fold increases in Aβ peptides produced in diabetic muscle. Consistent with those findings, *in situ *examination of diabetic and control muscle identified localized accumulations of each of these proteins in muscle sections from diseased animals, and also identified muscle fibers with central nuclei and evidence of cell infiltration.

## Methods

### Production of diabetes and hyperglycemia in rabbits

New Zealand white rabbits (Covance) were used in accordance with NIH guidelines and approved protocols. All rabbits were males to limit hormonal effects at this stage of our studies. 3-4 month-old rabbits were housed at RT° with a 12 hr light/12 hr dark cycle, and fed 260 gm/day of a standard pellet diet with free access to dH_2_O. Seven rabbits were given 150 mg/kg alloxan (Sigma) in a single injection through a catheter in an ear vein and six untreated rabbits were used as normal controls. During the initial 24 hrs after alloxan, 10% glucose in dH_2_O was given to reduce adverse effects. After one week, rabbits exhibiting a consistent elevation in blood glucose (> 350 mg/dl) remained in the study. Normal blood glucose in control rabbits was ~100 mg/dl, and blood glucose was monitored prior to feeding each morning. At the start of this study, rabbits weighed ~2.5 kg. After 16 wks, rabbits were taken for analysis. Diabetic rabbits gained an average of 225 gm (s.d. 180 gm) and control rabbits gained on average 500 gm (s.d. 50 gm), with no rabbits losing weight.

### Immunohistochemistry and Immunofluorescence

Paraffin sections were prepared from buffered 4% paraformaldehyde fixed muscle tissues. Sections were de-waxed in xylenes and graded alcohol washes, and blocked in PBS with 10% serum corresponding to the 2° antibody used. For immunofluorescence detection, fluor-conjugated 2° antibodies (Invitrogen) were used to visualize complexes. Immunohistochemical staining used horseradish peroxidase Vectastain kits (Vector labs). Antibodies included: mouse mAb anti-PrP (Cayman Chemical Co.), rabbit mAb anti-PrP (Epitomics), 6E10 and 4G8 mAb anti-Aβ (Covance), rabbit anti-AβPP (Sigma), sheep polyclonal anti-SOD1 (CalBiochem) and mouse mAb anti-SOD1 (Thermo), mouse mAb anti-NOS1 and anti-NOS2 (BD Biosciences).

### Immunoblot detection of proteins

Samples of total protein from quadriceps muscle from diabetic and normal control animals were homogenized in SDS sample buffer with reducing agent and protease inhibitors (Calbiochem), for electrophoresis on Bis-Tris gels (Invitrogen). Proteins resolved by molecular weight were blotted to PVDF filters, and blocked in 5% non-fat milk in PBS pH 7.4. Filters were probed overnight with antibodies diluted in PBS according to the supplier. HRP conjugated 2° antibodies (Jackson Labs) were used to visualize complexes with chemiluminescence kits on films (Amersham).

### ELISA assays

To quantify Aβ peptide levels in muscle tissue, ELISA kits (Covance) specific for Aβ peptides ending at amino acid 42 or amino acid 40 were used (x-42; x-40). These standard sandwich ELISAs used a primary antibody that recognized the Aβ peptides' C-terminus and distinguished Aβ40 from Aβ42, and the secondary antibody recognized an internal peptide epitope. Controls and standard curves were prepared as directed by the supplier. 150 mg of muscle tissue from experimental and control animals was solubilized in 5 M Guanidine buffer, and colorimetric quantification of HRP reaction product linked to antibody-antigen complexes was measured with a standard spectrometer plate reader. ELISA assays were performed three times for x-40 and x-42 Aβ peptides. A statistical power analysis indicated that three rabbits are theoretically required to provide sufficient power for this study, and this was also true when accounting for several fold differences we measured in our assays http://www.dssresearch.com/toolkit/sscalc/size.asp. For the ELISAs, the p-values were calculated using a 2-tailed ANOVA statistical test.

## Results

### PrP, Aβ, SOD1, NOS1 and NOS2 overall expression increases in total muscle protein samples from diabetic rabbits

**Figure 1:**
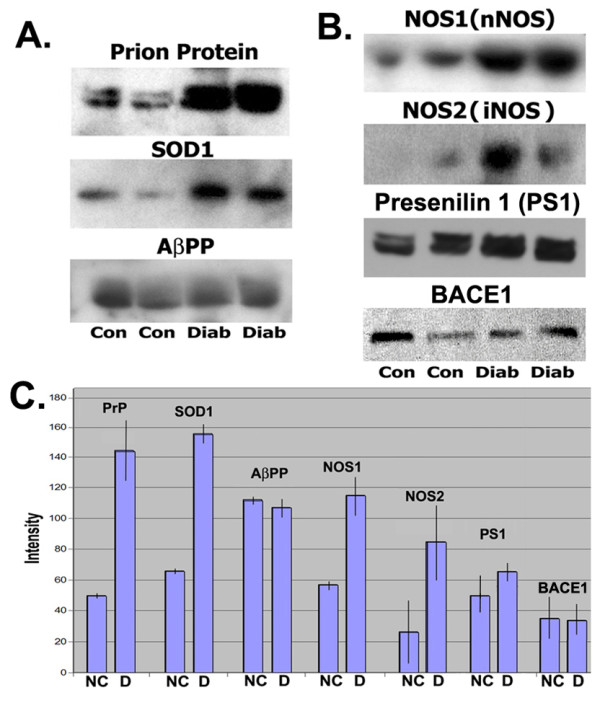
**Increased expression of the Prion Protein (PrP), Superoxide dismutase 1 (SOD1), and Nitric oxide synthases 1 and 2, but not the Alzheimer Aβ precursor protein (AβPP), Presenilin 1 or BACE1 occurs in total muscle protein from diabetic rabbits**. Immunoblot detection of proteins in diabetic and control muscle. **A: **PrP (MW ~30kDa, Epitomics), SOD1 (MW ~16kDa, Calbiochem), AβPP (MW ~110 kDa, Sigma), **B: **NOS1 (MW ~160 kDa, BD Biosciences), NOS2 (MW ~130 kDa, BD Biosciences), Presenilin 1 (MW ~52 kDa, Santa Cruz), BACE1 (MW ~55kDa, Santa Cruz) in total protein samples of quadriceps muscle from normal control (Con) and diabetic (Diab) rabbits. The doublet protein bands for PrP in diabetic and normal control muscle in Panel A is consistent with glycosylated forms, **C: **Densitometry measurements of protein bands shown on immunoblots above. Bar graphs show averages of the two band intensities with standard deviations. (NC: normal controls; D: diabetic).

Rabbits injected with alloxan became hyperglycemic and maintained blood glucose levels >350 mg/dl. After 16 weeks of elevated blood glucose levels, we took quadriceps muscle samples for analysis. We examined changes in overall expression of PrP, SOD1, NOS1, and NOS2, and for AβPP and Aβ peptide products in total muscle protein from diabetic and control animals. We first probed immunoblots with antibodies raised against PrP to determine if changes in expression occurred in diabetic muscle. mAb anti-Prion antibody (and rabbit polyclonal Ab; not shown) identified a significant increase in expression of PrP in diabetic muscle (Fig. 1A). These findings also agreed with *in situ *detection of PrP protein in muscle described below. We observed a protein doublet on immunoblots of total protein from experimental and control animals, and it appears to correspond with single glycosylated forms described in the literature [40]. Densitometric analysis of films after chemiluminescence detection of proteins indicated a ~3-fold increase in PrP expression occurred in diabetic muscle compared with normal controls (Fig. 1C). We next examined SOD1 protein expression in diabetic and control muscle, and detected >2-fold increase in SOD1 in muscle from diabetic animals on immunoblots (Fig. 1A), which was also consistent with our analysis of SOD1 accumulation described below. However, when we examined AβPP we did not see any significant changes in expression in normal *vs*. diabetic muscle (Fig. 1A). This finding was consistent with *in situ *analysis of AβPP protein expression (not shown), but differs from measurements of Aβ peptides produced in diabetic muscle described below. When we probed immunoblots with antibodies directed against the β-acting cleaving enzyme 1 and presenilin 1 protease that generate Aβ peptides, we did not observe substantial shifts in the levels of either protein, similar to our examination of amyloid and oxidative stress related PrP, SOD1, and NOS proteins in diabetic muscle. However, it is known that other factors including production of alternatively spliced AβPP isoforms [41] can influence Aβ levels.

Deleterious effects of nitric oxide in disease are thought to involve reactive NO species that can form *via *NO interactions with molecular oxygen or superoxide radicals [42]. In addition, deleterious increases in the level of NO also induce nitrosative stress and stimulate cellular pathways that contribute to protein S-nitrosylation and denitrosylation involving metalloproteins [43]. Increased expression and accumulation of Nitric oxide synthases 1 and 2 has been observed in muscle and brain diseases that are also associated with these amyloid proteins which are capable of producing oxidative stress [44]. When we examined changes in NOS1 and NOS2 protein levels in diabetic muscle on immunoblots, we identified a ~2-fold increase in NOS1 and NOS2 in total protein samples (Fig. 1B). NOS1 was detected more strongly in control muscle than NOS2, where NOS2 was barely detected in normal control muscle in our assays.

**Figure 2:**
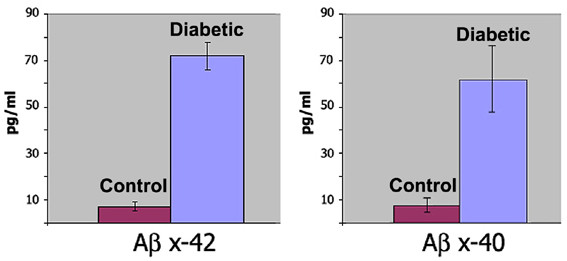
**ELISA assays measuring increased levels of Alzheimer Aβ peptides in diabetic muscle**. Levels of Alzheimer Aβ peptides ending at amino acid 40 (x-40) or 42 (x-42) were determined in total muscle proteins from three diabetic and three normal control rabbits. ~7.5-fold increases were measured for Aβ40 and Aβ42 peptides in diabetic muscle compared to normal control muscle (P < 0.0005 and P < 0.0025, respectively). Graphs show pg/ml of Aβ detected in muscle samples determined using standard peptide controls (Covance). Graphs show mean values of 3 diabetic and 3 control animals with standard deviations.

When we next examined Aβ peptide levels in normal and diabetic muscle, we measured quite substantial increases in Aβ peptides from diseased muscle using more quantitative ELISA methods. Aβ peptides are cleaved from the parent AβPP protein by β- and γ-secretases. Presenilin proteins are a key component of γ-secretase protease activity that cleaves Aβ at its C-terminus and is responsible for producing Aβ peptides ending at amino acid 40 or 42 [45]. In Alzheimer neurodegeneration, both Aβ 1-40 and 1-42 peptides increase in brain. However, Aβ 1-42 peptides are considered more deleterious in part due to greater metal affinity, which allows them to more readily form oligomers and produce hydrogen peroxide [23]. To measure Aβ peptides in diabetic muscle *vs*. normal controls, we used different ELISA assays that were specific for Aβ peptides ending at amino acid 40 or ending at 42. These assays do not detect the full length AβPP parent protein. Using these more quantitative methods, we measured ~7-fold more Aβ x-40, and a slightly higher increase of Aβ x-42 in muscle from diabetic animals compared to controls (Fig. 2). These increases also agreed with *in situ *detection using different Aβ specific antibodies, described below.

### Local accumulations of amyloid and oxidative stress-related proteins as well as disorganized fibers, occur in muscle in diabetic animals

**Figure 3:**
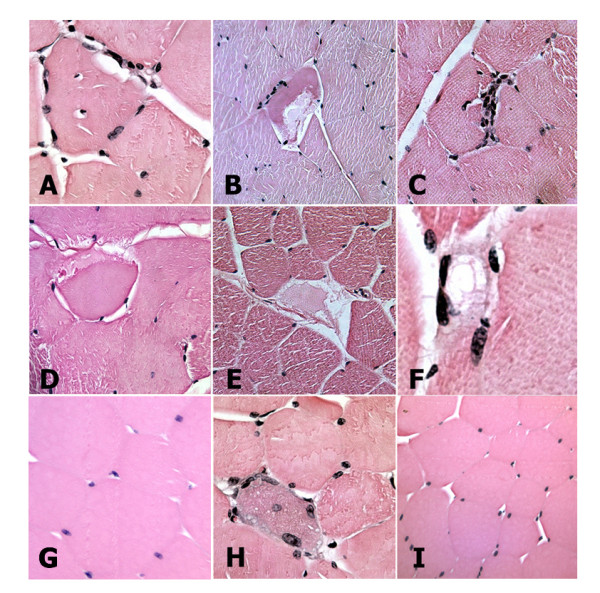
**Histological features generally characteristic of muscle degeneration linked with a variety of conditions are produced in quadriceps muscle from diabetic, hyperglycemic rabbits**. Hematoxylin and eosin stained muscle sections from diabetic (A-F, H) and control (G, I) rabbits demonstrating central nuclei (A, H) and showing evidence of cell infiltration (A, C, F), not seen in normal control muscle. Muscle fiber cross-sections are ~40-50 μm in diameter. Images are representative of 3 diabetic and 3 control animals.

**Figure 4:**
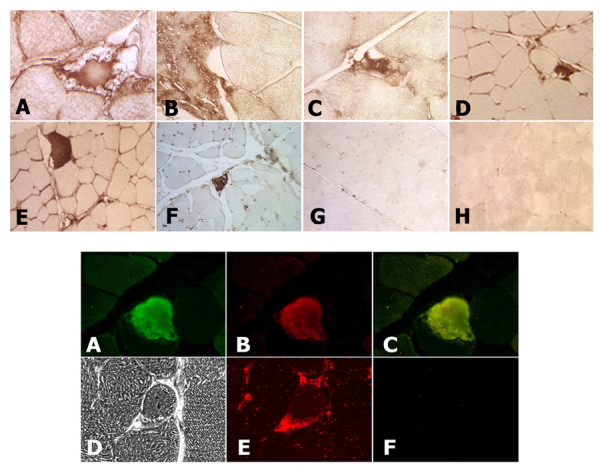
**PrP, Aβ peptides, SOD1, NOS1 and NOS2 protein deposits accumulate in muscle fibers in diabetic rabbits**. ***Above: ***Immunohistochemical peroxidase detection of **A: **Aβ peptides (mAb 6E10 Covance), **B, C: **PrP (rabbit mAb, Epitomics), **D: **NOS1 (mAb, BD Biosciences), **E: **NOS2 (mAb, BD Biosciences), **F: **Superoxide dismutase 1 (Calbiochem), **G: **No primary antibody, normal control, **H: **No primary antibody, diabetic muscle. ***Below: ***Immunofluorescence detection of proteins in diabetic muscle fibers. **A: **Aβ (mAb 4G8, Covance), **B: **PrP (mAb, Epitomics), **C: **overlay Aβ & PrP staining, **D: **Brightfield micrograph of degenerating muscle fiber showing vacuoles around the perimeter, **E: **Fluorescence photograph of anti-SOD1 staining in the same section as D, **F: **no primary antibody. Muscle fiber cross-sections are ~40-50 μm in diameter. Images are representative of 3 diabetic and 3 control animals.

Studies that examined muscle disease in animal models as well as in transgenic mice expressing amyloid and stress-related proteins in muscle, identified accumulations of these amyloid and oxidative stress proteins, predominantly with *in situ *methods. Similar protein deposits have also been observed in conditional transgenic mice induced to express these proteins in mature animals. We began our *in situ *analysis by examining fiber cell histology in hematoxylin and eosin stained sections. Regions in diabetic muscle contained disorganized fibers with swollen regions in muscle fibers (Fig. 3). In addition, muscle fibers were also observed that had central nuclei, and fibers surrounded by numerous hematoxylin stained nuclei suggestive of cell infiltration. When we probed sections with specific antibodies to determine the distribution of PrP, SOD1, Aβ and NOS1 and NOS2 in muscle from diabetic and control animals, we identified areas with localized accumulations for each of these proteins, consistent with the *in vitro *biochemical assays described above. We first probed sections with 6E10 and 4G8 monoclonal anti-Aβ antibodies (Fig. 4). These antibodies recognize epitopes in Aβ peptides that differ from those used in our ELISA assays. Both of these antibodies identified areas within muscle fibers that contained increased Aβ concentrations, and agreed with ELISAs. Likewise, muscle sections probed with antibodies raised against SOD1, PrP, NOS1, and NOS2 also identified regions with significant protein accumulation in muscle fibers from diabetic animals, also consistent with immunoblot assays (Figs. 4). In addition, when we probed muscle sections with antibodies to detect two of these proteins, we observed overlapping distributions of these proteins in muscle fiber deposits (Fig. 4).

## Discussion

In the present study we demonstrated that several classic amyloid and oxidative stress-related disease proteins increase in overall expression and produce localized accumulations in quadriceps muscle in diabetic rabbits in our wt animal model. We also identified histopathology that included the presence of central nuclei in muscle fibers and increased cell infiltration, also described in other muscle diseases and models. Our results indicate that stress produced during the onset and progression of diabetes and hyperglycemia induced in mature animals stimulates the expression of these proteins throughout the muscle. However, future studies are needed to determine the factors and processes involved in producing focal accumulations of these proteins in muscle fibers in diabetic animals. Our study determined that significant pathology was produced at 16 weeks in unmanaged diabetes and hyperglycemic animals in our model, and further studies can determine the relationship and onset of pathology associated with each of these proteins.

Our experiments that analyzed Aβ peptides used several antibodies that each identified increased production of Aβ in diabetic muscle. ELISA assays measured ~7-fold increases in Aβ peptides in total muscle protein samples. These assays used primary antibodies that recognized presenilin/γ-secretase cleavage sites at amino acid 40 or 42, and a common secondary antibody that recognized an epitope several amino acids from the Aβ C-terminus. As a result, these assays have the capability to measure a variety of peptide lengths that are cleaved at a γ-secretase site at one end. Antibodies used in our *in situ *Aβ analysis detected epitopes close to the N-terminus, and also identified increased Aβ in deposits in diabetic muscle. Although these antibodies can potentially detect the AβPP parent protein, we did not observe increased AβPP expression *in vitro *or in muscle sections with AβPP specific antibodies. Together, these findings indicate increased Aβ in diabetic muscle is largely the result of increased proteolytic Aβ cleavage. These observations are consistent with studies on Aβ production in the brain, where Aβ production in that organ has not been shown to correspond with significant changes in AβPP levels either. In addition, these observations in muscle are supported by findings in transgenic mice that showed transgenic co-expression of presenilin with AβPP in muscle also leads to enhanced Aβ production.

Each of the proteins examined here is linked with oxidative stress as well as with protein misfolding disease mechanisms. Studies of amyloid protein structure in neurodegenerative disease indicated these proteins can act synergistically to produce insoluble aggregates. For example, pathological changes in PrP structure can contribute to misfolding of other PrP proteins, and can also influence folding of nearby Aβ peptides in neurons [46]. The present finding of overlapping protein accumulations in diabetic muscle suggests similar interactions may also contribute to protein deposits containing multiple proteins in diabetic muscle. In addition, examination of the relative order and rate of production for each of these disease-related proteins at earlier stages after onset of diabetes and hyperglycemia in this model can also help sort out relative contributions of each protein in the formation of deposits in muscle fibers.

The present findings demonstrating a similar set of proteins accumulate in muscle fibers in this diabetes model as in other muscle disease conditions discussed above, is also supported by gene array studies that examined global gene expression changes in different models of muscle disease. Microarray experiments comparing expression profiles in muscle disuse, cachexia, repetitive stress, and diabetes in streptozotocin treated animals, found common changes in overall expression patterns and specific pathways in these disease models [47,48]. These observations also indicate similar pathways are triggered in a spectrum of muscle conditions. However, we speculate increased expression that produces substantial localized accumulations of Aβ, SOD1, PrP and NOS1 and NOS2 in a number of muscle disease conditions, suggests these proteins have a prominent and early role that is shared in muscle conditions linked with different modes of physiological stress. Increased NOS expression and NO production is linked with nitrative and nitrosative stress. Together with reactive oxygen species produced in non-enzymatic glycation reactions in diabetes, increased NO can react with superoxides to form peroxynitrite which is also a highly reactive oxidant and the increased intracellular production of NO and peroxynitrites can become cytotoxic [49-55]. The close relationship these proteins have with oxidative stress disease mechanisms suggests further that oxidative stress is also a common and early factor in these conditions. In summary, the present study demonstrates for the first time that pathophysiology resulting from diabetes and hyperglycemia substantially increases the overall expression of PrP, Aβ, SOD1 and NOS1 & 2 proteins in diabetic muscle, and produces localized accumulations of each of these proteins in muscle fibers.

## Conclusions

The present findings identify this well-characterized model of diabetes and hyperglycemia in wild-type rabbits as a new and potentially important venue for simultaneously reproducing a range of hallmark pathological features seen in a variety of muscle disease conditions linked with different etiologies. The production of diabetes and hyperglycemia in otherwise normal rabbits allows for normal physiological contributions by potentially synergistic disease mechanisms associated with amyloid and oxidative stress-related disease proteins analyzed here. Our demonstration of increased expression and localized accumulations of these proteins in diseased muscle that are more often associated with neurodegenerative disease also has the potential to inform about shared disease processes and potential therapeutics that can affect brain as well as muscle.

## Authors' contributions

CLB and PHF designed, performed and analyzed the data for the studies in this paper. YF assisted with experiments. NS provided assistance in discussing the data and assessing pathology in muscle samples. All authors read and approved the final manuscript.
